# Effect of deploying biomedical equipment technician on the functionality of medical equipment in the government hospitals of rural Nepal

**DOI:** 10.1186/s12960-022-00719-y

**Published:** 2022-03-04

**Authors:** Rita Thapa, Alison Yih, Ashish Chauhan, Salomi Poudel, Sagar Singh, Suresh Shrestha, Suresh Tamang, Rishav Shrestha, Ruma Rajbhandari

**Affiliations:** 1 Nick Simons Institute, EPC 1813 Sanepa, Box 8975, Lalitpur, Nepal; 2 Harvard Medical School, 25 Shattuck St, Boston, MA 02115 USA

**Keywords:** Assessment, BMET, Effectiveness, Functioning, Medical Equipment, Nepal

## Abstract

**Background:**

Medical equipment plays a crucial role in the provision of quality healthcare services, despite this more than 50% of equipment in developing countries are non-functioning due to a lack of appropriate human resources to maintain. To address this problem some government hospitals of Nepal have deployed a mid-level technical cadre called 'Biomedical Equipment Technician' (BMET). This study aims to evaluate the effectiveness of deploying a BMET on the functionality of medical equipment in government hospitals of rural Nepal.

**Methods:**

We used a mixed-methods approach with a comparative research design. A comprehensive range of 2189 pieces of medical equipment at 22 hospitals with and without BMET were observed to assess their functional status. Medical equipment were stratified into 6 categories based on department and *T* tests were conducted. We collected qualitative data from 9 BMETs, 22 medical superintendents, and 22 health staff using semi-structured interviews and focus-group discussions. Thematic content analysis was conducted to explore how the BMET's work was perceived.

**Findings:**

The quantity of non-functional devices in hospitals without BMETs was double that of hospitals with BMETs (14% and 7% respectively, *p* < 0.005). Results were similar across all departments including General (16% versus 3%, *p* = 0.056), Lab (15% versus 7%, *p* < 0.005) and Operation Theater (14% versus 5%, *p* < 0.005). Hospitals with BMETs had fewer overall non-functional devices requiring simple or advanced repair compared to hospitals without BMETs [3% versus 7% (*p* < 0.005) simple; 4% versus 6% (*p* < 0.005) advanced]. In our qualitative analysis, we found that BMETs were highly appreciated by hospital staff. Hospital workers perceived that having a BMET on staff, rather than twice-yearly visits from central-level maintenance technicians, is an effective way to keep medical equipment functional. However, without a favorable working environment, the BMET alone cannot perform optimally.

**Conclusions:**

Having a BMET at a rural government hospital has a substantial positive effect on the functional status of medical devices at the hospital. BMETs should be deployed at all rural hospitals to increase the functionality of medical devices, thereby improving the working environment and quality of health services provided.

**Supplementary Information:**

The online version contains supplementary material available at 10.1186/s12960-022-00719-y.

## Background

Sustainable maintenance of medical devices in government hospitals of low–middle-income countries (LMICs) is critical to efficiently delivering effective healthcare. Implications of malfunctioning medical devices include wasted healthcare funds, delayed times for patient treatment, and poorer healthcare outcomes for patients [1]. The World Health Organization estimated that over 50% of medical devices in LMICs are non-functional, not used correctly and optimally, and perpetually not maintained [2]. A survey carried out in Nepal showed that almost 61% of the medical devices of rural government hospitals were non-functioning due to a lack of an appropriate maintenance system [3]. Similarly, according to a hospital equipment need assessment conducted in rural Nepal in 2008, 81 out of 304 sampled devices required repair, which mostly required a simple fix [4]. Insufficient knowledge about operating devices, lack of maintenance experts, inappropriate referral systems for repair and lack of spare parts influence the availability, functionality, and utilization of equipment [5–7]. A systematic literature review revealed that funding for human resources to maintain medical equipment is a neglected area particularly in LMICs [8]. For example, a study of 5 sub-Saharan African countries demonstrated that less than half of hospitals assessed had equipment repair and maintenance services [9]. In response to the need for sustainable medical devices maintenance in rural Nepal, the National Health Training Centre (NHTC) of the Ministry of Health and Population (MoHP) of Nepal, adopted an innovative training program to develop technical human resources best suited for rural Nepalese hospitals—the Biomedical Equipment Technician (BMET) [10]. The BMET curriculum is a 48-week medical equipment technician training program that includes coursework in mathematics, science, laboratory maintenance, computer skills, and practical experience [10]. The course was upgraded to a Diploma Level Course (DBEE) in 2014. A service tracking survey conducted in 2018 showed that 48% (*n* = 43) BMETs were working in government hospitals [11]. Approximately, 245 BMETs have graduated as of 2020.

Some limited prior research has shown that deploying medical equipment technician educational programs have been effective for improving overall medical equipment functional status in hospitals of low-income countries [12, 13]. The BMET program, developed by Engineering World Health and deployed in Rwanda, Honduras, and Cambodia, resulted in significant decreases in non-functional equipment by 43%, 30%, and 30%, respectively, across 2 months in 2015 [12]. In addition, medical technician curricula not only benefit the maintenance of medical equipment, but also the technicians themselves. For example, a deployment of BMET in Rwanda resulted in technicians being 114% more productive than technicians that did not undergo BMET [13]. Many of these studies have been small in size and have not explored experiences of hospital staff working in hospitals with and without BMETs, nor perceptions and experiences of the BMETs themselves. Therefore, this study aims to evaluate the effectiveness of deploying a BMET on the functionality of medical equipment and also explore the perception, experiences, and satisfaction of hospital staff while working with and without BMET as well as analyze the perception, motivation, and satisfaction of the BMETs themselves while working in rural Nepalese hospitals.

## Methods

We used a mixed-methods approach, whereby we triangulated qualitative and quantitative data. For the quantitative component, we observed the functional status of medical equipment available in 11 government hospitals, where BMET was deployed and 11 government hospitals, where BMET was not deployed. The quantitative tool was developed based on the Government of Nepal certified "Minimum Service Standards" (MSS) checklist to evaluate the number of functional and non-functional equipment at the rural government hospitals [14]. We used part of the MSS checklist pertaining and non-functional equipment which was piloted in two non-study hospitals. Two external consultants with biomedical engineering degrees were hired who assessed all the equipment for its functional status and also attempts to fix the broken equipment during the study.

Qualitative data were collected simultaneously using semi-structured interviews and focus group discussions in all study areas. We were unable to collect qualitative data from 2 BMETs, because they were not at the hospital when we visited. Semi-structured interviews were conducted with 9 BMETs and 22 medical Superintendents. A total of 22 focus group discussions were conducted with hospital staff, including hospital storekeepers, who keep a record of the hospital equipment. There were 8 to 12 people in each FGD, and discussions took up 45–90 min in duration. Topic guides were developed in an iterative process throughout data collection to guide the discussion and interviews.

A team of trained Nepali field researchers collected qualitative data. These researchers were oriented to the purpose of the study and topic guides for 3 days before the field visits. Participants gave written informed consent to participate. All of the discussions were digitally recorded and transcribed and translated into English. To check the quality of translation, 5 pages of two randomly selected transcripts were back-translated into Nepali and compared with recordings.

### Sampling

Altogether, 22 government hospitals were sampled across all seven provinces of Nepal. In each province, districts were divided into three strata according to ecological zones. We used purposive sampling to select 11 districts with BMET first and then, 11 adjacent hospitals without BMET from the same ecological zone. The basis for match pairs was similar demographic contexts and access to outsourcing of biomedical equipment. The data were collected from December 2018 to February 2019.

### Data analysis

#### Quantitative

We analyzed the survey data descriptively and compared the functionality of equipment between hospitals with BMET and without BMET. The non-functional equipment category was further divided into those that required a “simple” fix versus those that required more “advanced” repair and referral. Other descriptive variables of data gathered on the government hospitals of rural Nepal included the number of hospital beds (15 or 50 beds), the presence of a maintenance department (yes or no), availability of spare equipment parts (“Not available,” “not sufficiently available,” or “sufficiently available”), the number of maintenance staff, and the quantities of each type of technician designation per hospital (BMET, BMEAT, or neither). Equipment was stratified by the following categories: Overall, General, Lab, Operation Theater, and X-ray. *T* tests were conducted on the quantitative results in Stata to compare whether differences between the number of functional equipment between the hospitals that had BMETs versus those that did not, were statistically significant.

#### Qualitative

A thematic content analysis was conducted, whereby the research team read a sample of the transcripts. A descriptive report of the preliminary analysis was written by one research team member, after which the research team independently generated themes from the data and came to a consensus through discussion. The data were coded according to themes in NVivo 10.

## Results

### Quantitative findings

A majority of the hospitals (64% of hospitals with BMET and 73% of hospitals without BMET) were 15-bedded hospitals, as shown in Table 1. Most (91%) of the hospitals, where BMETs were not deployed did not have any spare parts available for repair. In contrast, in hospitals, where BMETs were deployed, 27% of hospitals had some (but insufficient) spare parts available, and 36% had sufficient spare parts available. In addition, hospitals, where BMETs were deployed, had greater numbers of maintenance staff (usually 2 or more). These additional staffs were usually Biomedical Equipment Assistant Technicians (BMEATs) (82% in hospitals with BMETs versus 27% in hospitals without BMETs). Table 1:

**Table 1:** Characteristics of hospitals with BMET versus hospitals without BMET

Characteristic of Hospital	Hospital with BMET *n* (%)	Hospital without BMET *n* (%)
Number of Hospitals	11 (50%)	11 (50%)
Hospital Beds		
15 bedded hospitals	7 (64%)	8 (73%)
50 bedded hospitals	4 (36%)	3 (27%)
Availability of spare parts		
Not available	4 (36%)	10 (91%)
Partially available	3 (27%)	0
Sufficiently available	4 (36%)	1 (9%)
Availability of maintenance staff		
At least 1	11	3
Designations		
BMEAT	9 (82%)	3 (27%)
Neither BMET nor BMEAT	0 (0%)	8 (73%)

**Fig. 1:**
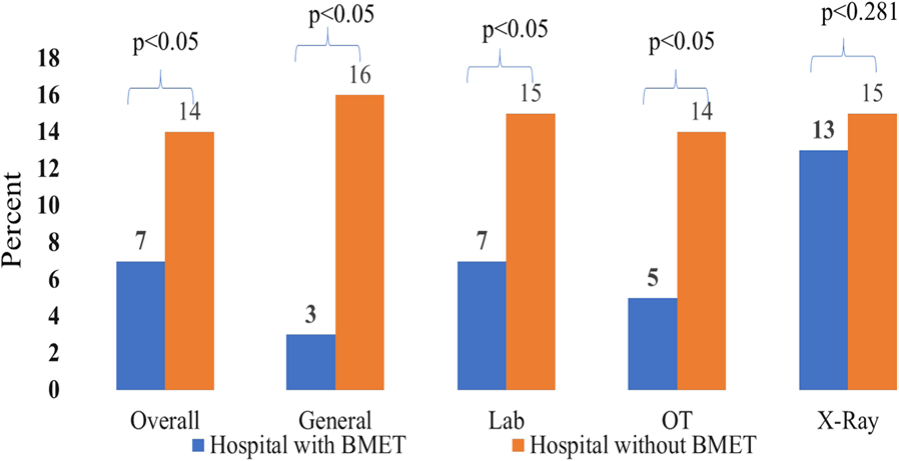
Non-functional equipment in various departments between hospitals with BMET versus hospitals without BMET

Per the results displayed in graph 1, the quantity of total non-functional equipment in hospitals without BMETs was significantly higher than in hospitals with BMETs in all departments in the hospital. For example, 14% of overall equipment was non-functional in hospitals without a BMET versus 7% in hospitals with a BMET (*p* < 0.005). Results were similar across all departments including General (16% non-functional equipment in hospitals without BMET versus 3% in hospitals with BMET, *p* = 0.056), Lab (15% versus 7%, *p* < 0.005) and Operation Theater (14% versus 5%, *p* < 0.005). The only department in which there was no statistically significant difference between hospitals with BMETs and those without was in the X-ray department. Although 15% of equipment in the X-ray department of hospitals without a BMET was defective versus 13% of equipment in the X-ray department of a hospital with a BMET, this difference was not statistically significant (*p* = 0.281) (Fig. 1).

According to Table 2, the quantities and proportions of non-functional equipment requiring advanced repair in hospitals with BMET were lower in all equipment categories. Differences between the hospitals with BMET and without BMET were significant for the overall category at 4% versus 6% (*p* < 0.005) and lab at 4% versus 7% (*p* = 0.021). The categories that were not statistically significant were for general equipment at 2% with BMET and 6% without BMET (*p* = 0.240), operation theater at 2% with BMET and 5% without BMET (*p* = 0.250), and X-ray at 11% with BMET and 12% without BMET (*p* = 0.637) (Table 2).

**Table 2:** Department wise non-functional equipment requiring advanced repair between hospitals with BMET versus without BMET

Department	Quantity of non-functional equipment requiring advanced repairment in hospitals with BMET (*n* = 1183)	Quantity of non-functional equipment requiring advanced repairment in hospitals without BMET (*n* = 1006)	*P*-value
Overall	48 (4%)	66 (6%)	< 0.005
General	8 (2%)	19 (6%)	0.240
Lab	12 (4%)	17 (7%)	0.021
Operation Theatre	4 (2%)	15 (5%)	0.250
X-ray	7 (11%)	6 (12%)	0.637

As shown in Table 3, all quantities and proportions of non-functional equipment that required simple repairs were lower in hospitals with BMET compared to hospitals without BMET. The results were significant for overall equipment at 3% versus 7% (*p* < 0.005) and X-ray equipment at 2% versus 4% (*p* = 0.009). The results that were not significant were for general equipment (1% for hospitals with BMET and 9% for hospitals without BMET, *p* = 0.096), lab (3% for hospitals with BMET and 8% for hospitals without BMET, *p* = 0.051), and operation theater (4% for hospitals with BMET and 8% for hospitals without BMET, *p* = 0.080) (Table 3).

**Table 3:** Department wise non-functional equipment requiring simple repair between hospitals with BMET versus without BMET

Department	Quantity of non-functional equipment requiring simple repairment in hospitals with BMET (*n* = 1183)	Quantity of non-functional equipment requiring simple repairment in hospitals without BMET (*n* = 1006)	*P*-value
Overall	32 (3%)	74 (7%)	< 0.005
General	6 (1%)	28 (9%)	0.096
Lab	7 (3%)	21 (8%)	0.051
Operation Theatre	11 (4%)	19 (8%)	0.080
X-ray	1 (2%)	2 (4%)	0.009

## Qualitative findings

### Availability of medical equipment

Respondents working in hospitals without BMET were struggling to provide health services due to a lack of functional medical equipment. One of the hospital staff expressed*, "We do not have an adequate number of equipment and also, most of the equipment we have is broken so we cannot use it to its full potential.”* In addition, they also felt that equipment was often dumped in the storeroom even if the problem or repair needed was minor. In addition, most of the BMETs also shared their experiences about the devasting situation of medical devices when they first reached their designated hospital, "… *the situation of the hospital was miserable. The equipment was not functioning. Some of the new equipment was not in use due to lack of knowledge about how to install them."*

In contrast, most of the staff working in hospitals having a BMET shared their experience that due to the ability to have instant repair by the BMET as well as the availability of spare parts and equipment, they rarely encountered problems of health service interruption. *"We usually hear in the news that the services of the hospitals have been interrupted due to damage to the X-ray and other machines in A or B hospitals but such things never happen in this hospital."* They also shared their experience that such a positive situation was not always the norm, "*Before the BMET was hired, we had a lot of problems. We had to refer patients when the X-ray machine [medical device] was not working. Infants did not get drugs due to the malfunctioning of the nebulizer machine. It is easy to work now."*

### Equipment repair mechanism

The qualitative data showed that there are different levels and mechanisms by which equipment is repaired at both hospitals with and without BMET. Equipment is usually maintained and repaired at the local and central levels. In general, hospitals themselves decide on the appropriate mechanism as needed.

#### Local-level repair mechanism

Data showed that all the hospitals with BMET follow a systematic process to repair medical equipment. Technicians are informed about the broken status of equipment by the hospital staff and then attempt to repair the damaged equipment. If it is beyond their capacity, they consult with their mentors at the central level or refer the equipment to a higher level. One of the staff working in a hospital with a BMET mentioned that *"Usually, if any machines such as the Blood Pressure set are damaged, I send that to the BMET and it is repaired immediately. One day, there was a problem with the plaster cutter. I sent that to BMET and he fixed it… and finally, I was able to cut that plaster."*

However, some of the BMETs working in hilly and mountainous areas expressed that due to a lack of spare parts, sometimes repairs may get delayed, *"Some equipment can be repaired instantly with a small amount of effort. However, due to the unavailability of spare parts, sometimes it takes a long time to get repairs done."*

In contrast, hospitals without BMET have a longer process to repair broken equipment and it usually takes a long time to get equipment repaired. Hospital staff stated that *"We can call the person from outside to repair equipment only if we have the budget for repair and maintenance. We have repaired just a few pieces of equipment. Most of the broken equipment is dumped when even a small repair could return them to a functional status."*

#### Central level repair mechanism

The Central Government has been managing the repair and maintenance of medical equipment with the coordination of external contractors over the past 4 years (2017 to 2021). Most of the respondents said that central level technicians come to their hospital once every 6 months to repair broken equipment. However, some of the respondents working in hospitals without BMETs in the Mountainous and Hilly regions complained that this frequency was just too low—*"When we had a problem with the X-ray machine, I informed the Medical Superintendent immediately. Then he called the central level technician team. They said that they would send their team members within 2–3 days but it has been 5 months and they have yet to arrive.”*

### Benefits of deploying BMET

Respondents have mentioned various benefits of having a BMET in the hospital. Healthcare workers from the hospitals having BMET said that their confidence level has increased while providing health services. Staff working in the Operation Theater mentioned, “*In the OT when there is a BMET, we feel so confident. We can tell him if any equipment is malfunctioning. BMET fixes the cautery machine any time during the procedure if there is any problem. So, there is a difference when we have him…All are confident*.” In addition, BMETs were also valued for their contribution in reducing the economic burden on hospitals, *"If a BMET were here, he would have been involved in the continuous maintenance of the machines…We generally have the trend of buying new equipment when something is damaged. We don’t pursue the repair of that equipment. In this sense, BMETs save money for the hospital."*

### BMET deployment process

Even though almost all the respondents agreed on the necessity of a BMET in rural government hospitals, regardless of size, that is critical to improve the functional status of medical equipment and provide uninterrupted quality healthcare services, half of the studied hospitals had not hired a BMET as the government has no established posts for BMETs in 15-bedded hospitals. According to the respondents, only hospitals with 50 beds have sanctioned posts for a BMET in the government organogram. Due to such criteria, most of the hospitals have not hired BMET through the government but rather through the support of External Development Partners (EDPs). One Medical Superintendent said, "*I think the planners and policymakers might have thought that BMET is not needed in this small hospital."*

### Working environment for BMET

There were mixed reactions from BMETs about their working environment at rural hospitals. Most of the hospital staff including the Medical Superintendent. have claimed to be supportive of their BMET, making the working environment suitable for the BMET to work in, "*We have been encouraging him to do the work. For example, when I call him for the repair of machines if the problem is urgent, then he comes immediately. If I can manage the problems and run the service for some time, then I tell him to come later for repair. In this sense, I have made it easier for him to work."*

A few BMETs reported good support from colleagues, "*They are helpful and supportive. The information flow is good. They have informed me about the equipment available in the store."* In contrast, some of the BMETs were not fully satisfied, "*I worry because sometimes they do not inform me even if the equipment has been non-functional for 1–2 months."*

Most of the BMETs were worried about retaining their technical knowledge and skills due to limited equipment available at rural government hospitals—*"I have been working here for 3 years and there is not much-advanced equipment. I am scared that if we just deal with simple equipment then I may not retain my skills to repair advanced equipment."*

### Discussion

This study evaluates the effect of deploying BMETs on the functional status of medical equipment in rural government hospitals of Nepal. We found that having a BMET has a substantial positive effect on the functional status of equipment at a rural hospital.

The hospitals where BMETs were deployed had fewer non-functioning equipment that required simple and advanced repairs in all categories of equipment (general, lab, operation theater, and X-ray), compared to government hospitals without a BMET. Significant differences between hospitals with BMET and hospitals without BMET in the total number of non-functional equipment were found in the overall, lab, and operation theater categories (*p* < 0.005). For non-functional equipment that required advanced repair, the differences between hospitals with and without BMET were statistically significant in the overall (*p* < 0.005) and lab categories (*p* < 0.05). For non-functional equipment that required simple repair, the differences between hospitals with and without BMET were statistically significant in the overall (*p* < 0.005) and X-ray categories (*p* < 0.05).

Our results are consistent with other studies in the literature showing reductions in the number of non-functional equipment in hospitals, where BMETs were deployed. For example, in a 2015 study by Emmerling et. al., focused on Honduran hospitals, BMET deployment resulted in reductions in the quantity of non-functional equipment by 43%, 30%, and 30% for each hospital studied (*p* < 0.0001) [12]. In addition, technicians who trained in the Biomedical Technician Assistant (BTA) program of Rwanda were able to repair 79% of equipment in the hospitals they were deployed in [15]. In another study in Rwanda, non-BTA hospitals had 54% more non-functional equipment than BTA hospitals [16].

Limitation of resources is a major constraint that medical equipment technicians at the rural hospitals of lower and middle-income countries continue to face. Despite this, Malkin & Keane found that of sixty resource-poor hospitals from 11 countries, 66% of medical equipment was able to be restored to functioning order with the intervention of the BTA program [17]. BTA technicians had only 70.8% of unrepaired equipment compared to non-BTA technicians having 85.0% of unrepaired equipment attributed to the scarcity of spare parts in Rwanda hospitals (*p* < 0.25) [17]. Resource scarcity was also present in the sampled government hospitals of Nepal in this study, as shown in Table 1, since only 36% of hospitals with BMET and 9% of hospitals without BMET reported the stock of spare parts as “sufficiently available.” However, there was a statistically significant difference between the number of total non-functional equipment between rural hospitals with and without BMET, as shown in Table 2: 7% of equipment from rural hospitals with BMET and 14% of equipment from rural hospitals without BMET were not functional (*p* < 0.005).

As shown in Gammie’s 2012 study, performing simple fixes on medical equipment can mitigate the need for advanced fixes [4]. Such observation was demonstrated in our study as well as shown in Table 3: 3% of overall equipment in hospitals with BMET and 7% of overall equipment in hospitals without BMET required simple repair (*p* < 0.005). 4% of overall equipment in hospitals with BMET and 6% of overall equipment in hospitals without BMET required advanced repair (*p* < 0.005). The reduced quantities of non-functioning equipment can be attributed to BMET being more informed and capable of preventive maintenance.

Most of the respondents from the qualitative portion of our study criticized the mechanism of equipment repair from the central level as it takes a long time and there is a high probability of equipment losses in cases where the equipment has to be sent out of the hospital. Rather, healthcare workers preferred to have a local BMET who could repair equipment immediately, as soon as it was broken, and thereby be a source of confidence in providing continuous and quality health services.

A tracking survey conducted in 2018 showed that 43 BMETs [11] were hired by government hospitals, and half of them have permanent (government) posts which indicates that the importance of deploying BMET in rural hospitals has been internalized by the concerned stakeholders at the central and provincial levels. However, due to certain criteria like a permanent sanctioned post for a BMET at only 50-bedded hospitals (not 15-bedded hospitals), some of the hospitals have not been able to deploy a BMET, even though they urgently need one [11].

Although this study did not perform an economic evaluation, the experiences of hospital staff revealed that having a BMET enabled broken equipment to be repaired in a cheaper and quicker manner than would have occurred without the BMET. This finding is congruent with previous literature suggesting an overall element of cost-effectiveness in expanding human resources for health [18–21]. Similarly, the confidence level of health workers working in hospitals with a BMET is higher, because they can provide services without the pressure of equipment malfunctions. Favorable working environments consisting of team support and adequate availability of spare parts enabled BMETs to work to their full potential.

### Limitations

This study had several limitations. The data did not capture the geographic origin and brand of manufacture for each piece of medical equipment surveyed, which can affect equipment functionality outcomes related to lack of warranty and inadequate understanding of low–middle-income countries’ medical equipment needs [22, 23]. Geographic origin can also play a role in whether equipment is used or ultimately discarded [24–26]. This is exacerbated by how only 13% of medical equipment manufacturers are based in low–middle-income countries, thus increasing the dependency on imported equipment and spare parts [25]. In addition, stakeholders may also have responded positively to continue to receive the benefits of the BMET program from NSI although we assured respondents that NSI could not access the raw data, and the study used an independent consultant to facilitate discussions, to try to minimize this bias.

### Conclusions

In conclusion, we found that having a BMET at a rural hospital has a substantial positive effect on the functional status of equipment at the hospital. The hospitals where BMETs were deployed had fewer non-functioning equipment that required simple and advanced repairs in all categories of equipment compared to government hospitals without a BMET. This study suggests that BMETs should be deployed at all rural hospitals in Nepal and other low- and middle-income countries to increase the functionality of medical equipment, thereby improving the working environment and quality of health services provided at these hospitals. Future studies need to be conducted in the long term to evaluate the financial sustainability of deploying BMETs (Additional file 1).

## Supplementary Information

**Additional file 1.** Reference detail with web links.

## Data Availability

The qualitative and quantitative data used for this study are available and the corresponding author is responsible to provide it if there is a reasonable request.

## Abbreviations

BMET: Biomedical Equipment Technician
BMEAT: Biomedical Equipment Assistant Technician
DBEE: Diploma in Biomedical Equipment Engineering
MoHP: Ministry of Health and Population
MSS: Minimum Service Standard
NHTC: National Health Training Center
LMIC: Low–middle income country
