# Supplementary figures and images for: Effect of deploying biomedical equipment technician on the functionality of medical equipment in the government hospitals of rural Nepal
Source: Hum Resour Health. 2022 Mar 4;20:21. doi: 10.1186/s12960-022-00719-y (PMC8895523; doi:10.1186/s12960-022-00719-y)

Location of study sites


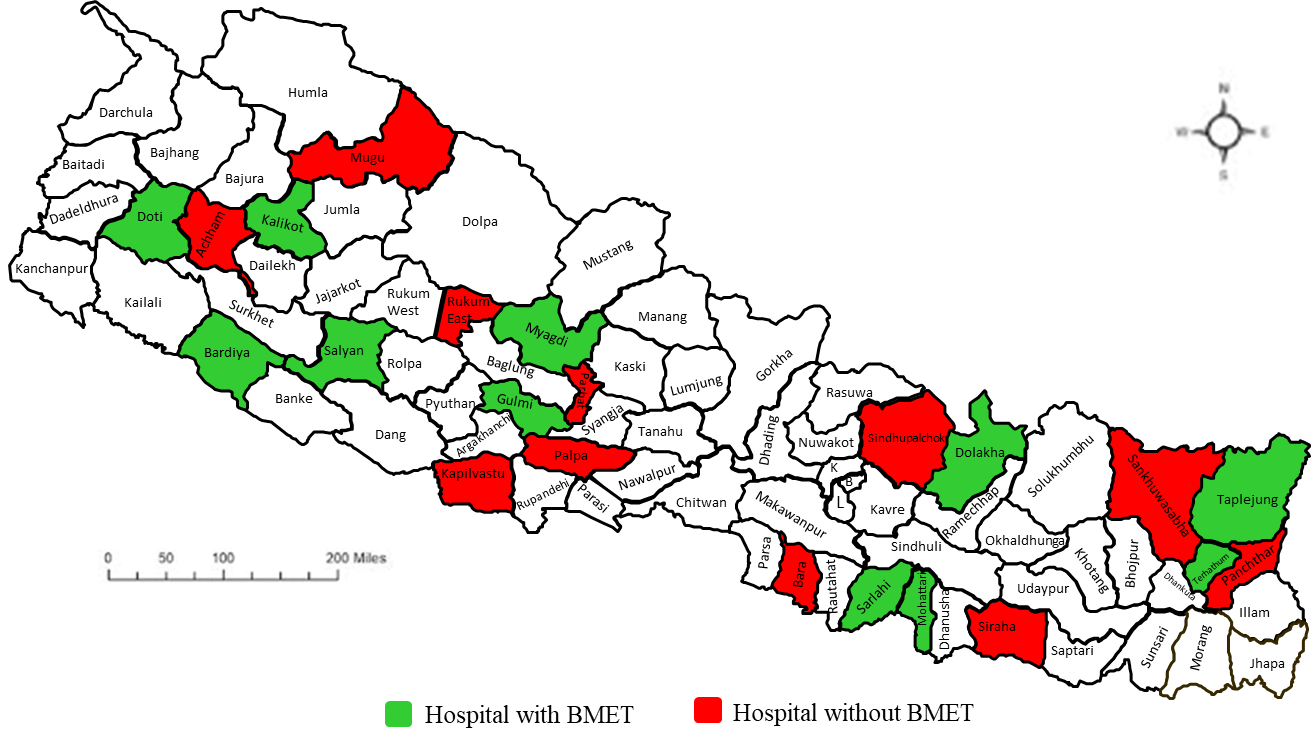

Supplement: Supplementary file 1 — Additional file 1. Reference detail with web links. [file 12960_2022_719_MOESM1_ESM.docx]
